# Profiling Inflammatory Responses with Microfluidic Immunoblotting

**DOI:** 10.1371/journal.pone.0081889

**Published:** 2013-11-27

**Authors:** Huai-Ning Chang, Pascale R. Leroueil, Katherine Selwa, C. J. Gasper, Ryan E. Tsuchida, Jason J. Wang, Walker M. McHugh, Timothy T. Cornell, James R. Baker, Sascha N. Goonewardena

**Affiliations:** 1 Michigan Nanotechnology Institute for Medicine and Biological Sciences, University of Michigan, Ann Arbor, Michigan, United States of America; 2 Division of Cardiovascular Medicine, Department of Internal Medicine, University of Michigan, Ann Arbor, Michigan, United States of America; 3 Division of Pediatric Critical Care Medicine, Department of Pediatrics and Communicable Diseases, University of Michigan, Ann Arbor, Michigan, United States of America; University of California, Merced, United States of America

## Abstract

Rapid profiling of signaling pathways has been a long sought after goal in biological sciences and clinical medicine. To understand these signaling pathways, their protein components must be profiled. The protein components of signaling pathways are typically profiled with protein immunoblotting. Protein immunoblotting is a powerful technique but has several limitations including the large sample requirements, high amounts of antibody, and limitations in assay throughput. To overcome some of these limitations, we have designed a microfluidic protein immunoblotting device to profile multiple signaling pathways simultaneously. We show the utility of this approach by profiling inflammatory signaling pathways (NFκB, JAK-STAT, and MAPK) in cell models and human samples. The microfluidic immunoblotting device can profile proteins and protein modifications with 5380-fold less antibody compared to traditional protein immunoblotting. Additionally, this microfluidic device interfaces with commonly available immunoblotting equipment, has the ability to multiplex, and is compatible with several protein detection methodologies. We anticipate that this microfluidic device will complement existing techniques and is well suited for life science applications.

## Introduction

Inflammation is now recognized as a driver of several chronic diseases including cancer and heart disease[1,2]. Although many regulatory steps are involved, protein modifications are one of the defining features of inflammatory responses[3-5]. Since its inception in 1979, protein immunoblotting has become the standard technique for profiling proteins and protein modifications in molecular biology and clinical diagnostics[6]. Although traditional protein immunoblotting is a powerful technique, it has several limitations including its slow throughput, the requirement for relatively large sample and antibody amounts, and the inability to probe for multiple proteins simultaneously[7]. 

As our recognition of the role of inflammation in disease has grown, there is a need for more robust approaches to monitor the signaling pathways that drive inflammation. To overcome some of the limitations of traditional protein immunoblotting, several variations have been introduced including membrane stripping and the use of fluorescent secondary antibodies. Despite their improvements, these variations have their own limitations, including loss of signal intensity and increased assay variability. Additionally, none of these variations address the large sample and antibody amounts required by traditional protein immunoblotting. 

Recently, microfluidic technology has been applied to molecular biology and in clinical diagnostics. The small volumes and spatial control afforded by microfluidics make it an exciting complement to existing technologies. With regards to protein immunoblotting, microfluidic immunoblotting devices have been fabricated with most approaches trying to integrate all aspects of protein immunoblotting. The Herr group has recently presented several approaches that incorporate microfluidic technology with traditional protein immunoblotting[8,9]. Their approaches are significant improvements over existing immunoblotting platforms because of the integration of all aspects of protein immunoblotting [10]. Pan and colleagues made advances by introducing a fluorescence based microfluidic immunoblotting device that could detect several proteins and is compatible with existing protein immunoblotting technologies[11]. We wished to build upon these advancements without sacrificing the accuracy and accessibility of traditional protein immunoblotting. We fabricated a microfluidic device that can simultaneously profile multiple proteins using existing immunoblotting equipment and chemiluminescent detection technologies. We demonstrate the utility of this microfluidic device by monitoring several inflammatory signaling pathways in culture models and human samples. 

## Materials and Methods

### Microfluidic protein immunoblotting

The transparency mask was printed using a CAD file of the microfluidic device (CAD/Art Services, Inc.; Bandon, OR USA). Soft-lithography was used to fabricate a silicon master mold from the transparency mask. The microfluidic devices were created by mixing the pre-polymer and curing agent (Sylgard 184 Silicone Elastomer Kit, Dow Corning; Midland, MI USA) and then pouring on to the silicon master. 

After gel electrophoresis, the gels were electroblotted onto a PVDF membrane (Life Technologies; Carlsbad, CA USA) for 1 hour at 30V. The PVDF membrane was dried overnight before proceeding to microfluidic immunoblotting. The microfluidic assembly was created by sandwiching the PVDF membrane between a glass support and the PDMS microfluidic device. The PDMS microfluidic device was aligned to the ladder to ensure accurate channel placement over the sample lanes. To complete the assembly, two glass slides were placed on top of the PDMS device for further support. The spacing between the two glass slides was approximately 0.5mm and was used as the injection site. The activating and antibody solutions were injected using a 1ml syringe with 27G ½ needles. The PVDF membrane was activated through the microfluidic channels using a 0.1% BSA and 0.1% Tween20 in TBS. Primary antibodies were injected through the microfluidic channels and incubated for 1 hour. Both the primary and secondary antibody dilutions were prepared in 0.1% BSA and 0.1% Tween20 TBS. Following the primary antibody incubation period, the microfluidic device was removed and the membrane was washed, blocked for 1 hour in 0.1% BSA and 0.1% Tween20 TBS, and then stained with the secondary antibody for 1 hour prior to chemiluminescent detection with either alkaline phosphatase or horseradish peroxidase.

### Ethics Statement

For peripheral blood mononuclear cells (PBMCs), healthy individuals who agreed to participate in this study provided written informed consent. The study was approved by the Institutional Review Board at the University of Michigan, HUM00049322. 

### Gel electrophoresis and protein immunoblotting

PBMCs were isolated using BD Vacutainer CPT Cell Preparation Tubes with Sodium Citrate. Following isolation, PBMC lysates were collected as described below. The murine macrophage cell line (RAW264.7) was purchased from American Type Culture Collection (ATCC; Manassas, VA USA) and cultured in RPMI medium (Life Technologies; Carlsbad, CA USA) supplemented with penicillin (100 U/ml), streptomycin (100 μg/ml) and 10% fetal bovine serum (Invitrogen) at a density of 3.0 X 10^6^. After the cells were allowed to adhere for 4 to 6 hours, the media was changed to RPMI media supplemented with 0.5% FBS, penicillin (100 U/ml), streptomycin (100 μg/ml), and incubated overnight (14-18 h). Endotoxin stimulation was initiated by incubating cells in 0.5% FBS medium containing lipopolysaccharide (LPS) from *Escherichia coli* O55:B5 (Sigma-Aldridge; St Louis, MO USA) at a concentration of 100 ng/ml. Cells were then harvested and lysed using RIPA buffer containing 25 mM Tris-HCl, pH 7.6, 150 mM NaCl, 1% Nonidet P-40, 1% sodium deoxycholate, 1 mM Na_3_VO_4_, and 10 mM NaF (phosphatase inhibitors) and 0.1% SDS with 10 µl of Halt Protease Inhibitor Cocktail (Pierce; Rockford, IL USA) added for each 1 ml of buffer. Samples were separated using SDS-PAGE electrophoresis and transferred to a PVDF membrane. PVDF membranes were probed with rabbit primary antibodies against active-JNK (phospho-JNK1/2; Promega; Madison, WI), active-MAPK (phospho-ERK; Promega), and ERK-1 (Santa Cruz Biotechnology; Santa Cruz, CA USA), NF-κB p65 (Cell Signaling Technology; Beverly, MA USA), STAT3 (sc-483, Santa Cruz Biotechnology; Santa Cruz, CA USA), and phospho-STAT3 (Cell Signaling Technology; Beverly, MA USA). Horseradish peroxidase (HRP) or alkaline phosphatase (AP) goat anti-rabbit secondary antibodies (Thermo Scientific; Rockford, IL USA) were used at a dilution of 1:20000.

### Quantification and statistical analysis of protein immunoblots

Immunoblots were scanned and converted to 2400 dpi images. The images were imported into ImageJ software and analyzed as described below. For the traditional protein immunoblots, the signal intensity inside three boxes overlaying the band of interest was measured (each box was 10 pixels x 28 pixels); the average of these measurements was the signal intensity assigned to the band. The standard deviation of the signal intensity inside the three boxes was on average 9.9%. For the microfluidic protein immunoblots, the signal intensity of overlaying the region of interest was measured (each box was 10 pixels x 28 pixels). For both traditional and microfluidic immunoblots, the signal intensity was normalized to the signal intensity from the 5 µg of protein probed with the 1:1000 dilution of P65 antibody. Traditional and microfluidic immunoblots are representative of at least three independent protein immunoblots. Signal quantification is expressed as means with standard deviations (SD) with numbers of individual experiments presented in figure legends. Significance was tested using analysis of variance (ANOVA) with Newman–Keuls post hoc test (P<0.05). 

## Results

### Design and interface of the microfluidic device

The poly(dimethysiloxane) (PDMS) microfluidic device was designed to interface with existing immunoblotting equipment. The devices were fabricated using standard soft-lithography techniques as previously described[12].First, the transparency mask was printed from a CAD file of the microfluidic device. Soft-lithography was used to fabricate a silicon master mold from the transparency mask. The microfluidic device consisted of 5 lanes corresponding to the protein lanes of a traditional protein gel. Each lane contained 3 microfluidic channels (3.1 cm long, 150 μM wide, 100 μM deep). The microfluidic devices were created on the silicon master by mixing the pre-polymer and curing agent at a mass ratio of 12:1. The microfluidic device was degassed and cured at 60° C for 1 hour. 

**Figure 1:**
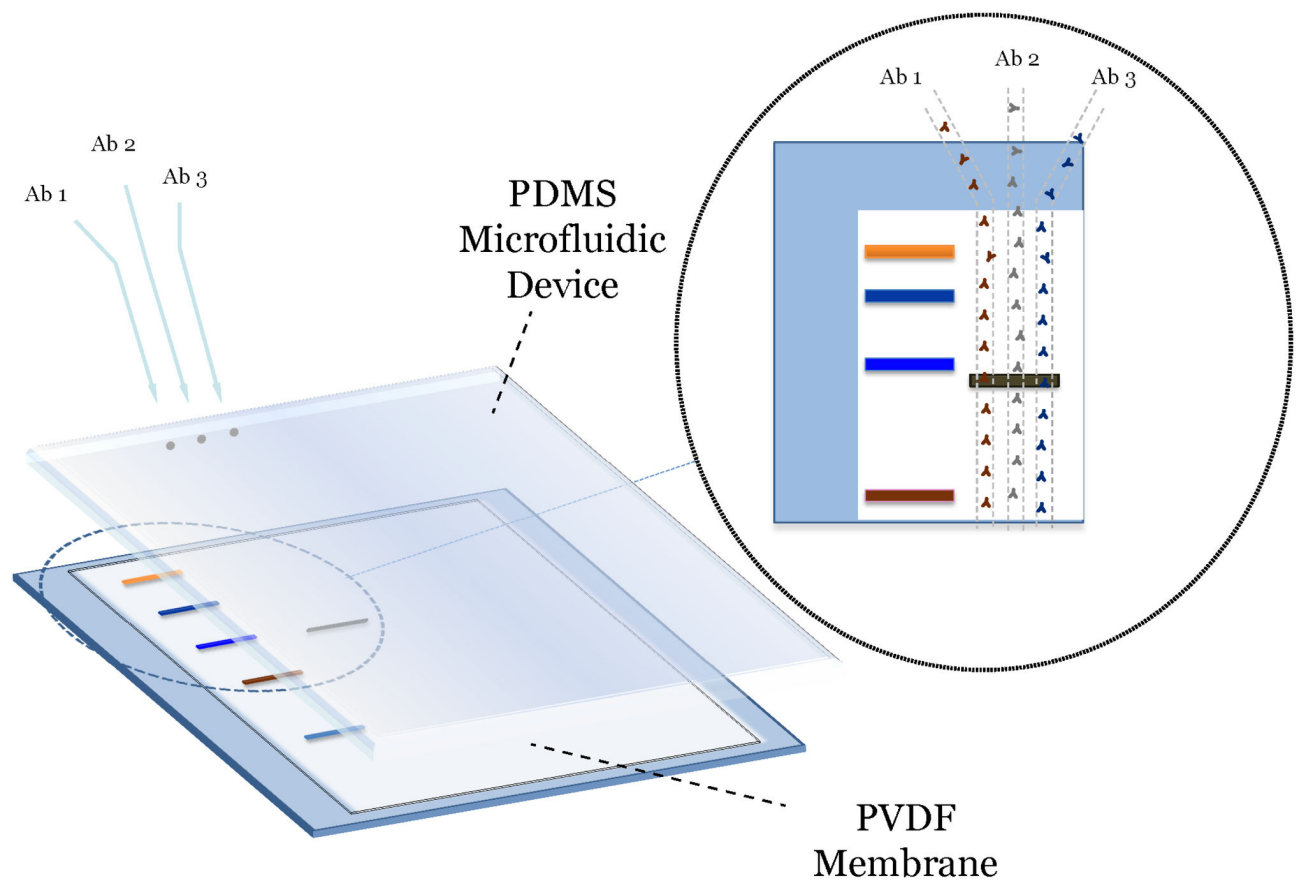
Schematic of a PDMS microfluidic device and the interface with a PVDF membrane. Microfluidic channels overlie each sample lane that can be used to probe for multiple proteins within each sample lane.

Our microfluidic immunoblotting approach consists of three stages including gel electrophoresis, transfer to a PVDF membrane, and microfluidic immunoblotting for proteins of interest (Figure **1**). Because the first two stages are similar to traditional immunoblotting, the microfluidic device easily interfaces with existing immunoblotting equipment. After the samples were separated and transferred to the PVDF membrane, the PVDF membrane was placed between a glass support and the microfluidic device. The PVDF membrane was activated by filling and incubating the microfluidic channel with blocking solution for 20 minutes. After the PVDF membrane was activated, the microfluidic channels were filled with the primary antibody and incubated for 1 hour. The microfluidic device was then removed and the PVDF membrane was washed. To detect the primary antibody, the entire PVDF membrane was incubated with the chemiluminescent secondary antibody and detected with the specified chemiluminescent substrates. 

### Comparison of traditional and microfluidic immunoblotting

**Figure 2:**
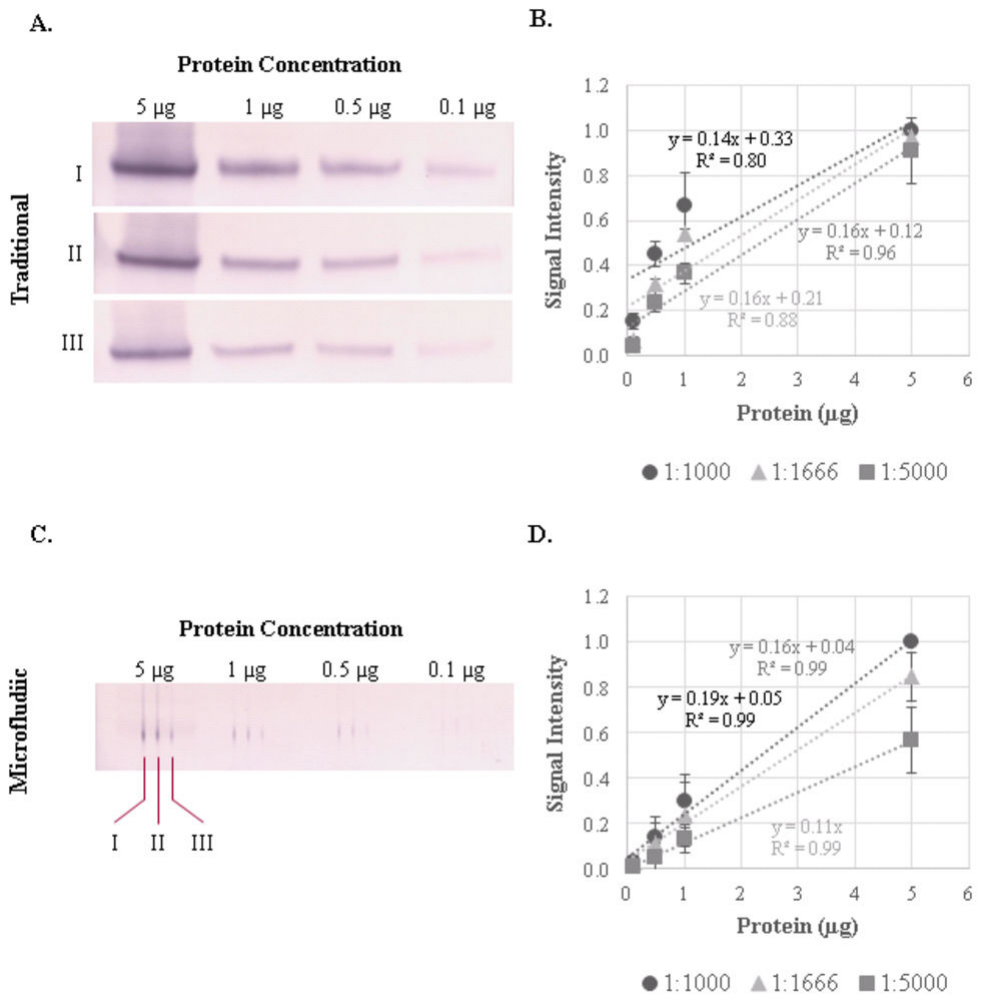
Comparison of traditional and microfluidic immunoblotting in human blood monocyte samples. Representative immunoblots for RelA/p65 at four protein concentrations (5, 1, 0.5 and 0.1 µg) and three antibody dilutions (I: 1:1000; II: 1:1666; III: 1:5000) using (A) traditional and (B) microfluidic immunoblotting techniques. The signal intensity for the (C) traditional and (D) microfluidic blots were quantified using ImageJ software and normalized to the signal associated with 5 µg of protein probed with the 1:1000 p65 antibody dilution. Immunoblots are representative of three independent PVDF membranes from the same PBMC lysates.

We wished to compare the test characteristics of microfluidic immunoblotting with traditional immunoblotting. We collected peripheral blood monocytes (PBMC) from healthy volunteers and isolated protein lysates. Four different protein amounts were separated using gel electrophoresis and then transferred to a PVDF membrane. Using a primary antibody to RelA/p65, a member of the NF-κB transcriptional family, we compared the signals between microfluidic and traditional immunoblotting at three different primary antibody dilutions and the different protein loading amounts (Figure **2** and Figure **S1**). The microfluidic immunoblotting approach resulted in slightly lower, but comparable signal intensities compared to those from traditional immunoblotting. In both approaches, the signals and signal variation were comparable under the conditions tested. 

For the PVDF membranes used in these experiments, a traditional immunoblotting assay for a single protein requires approximately 10 mL of primary antibody solution. Based on the microfluidic channel (0.015, 0.010, and 3.100 cm), approximately 0.000465 mL of primary antibody solution is required for each microfluidic channel. Using the microfluidic device, to assay a PVDF membrane for 1 protein requires 0.00186 mL of primary antibody solution (4 lanes *0.000465 mL of antibody/lane = 0.00186 mL primary antibody solution). Even without considering the multiplexing capacity of the microfluidic device, this translates into a 5380-fold reduction in primary antibody amounts compared with traditional protein immunoblotting. The implication of these results is that the microfluidic approach requires substantially less protein and antibody amounts to generate immunoblots of comparable quality. To further demonstrate the multiplexing ability of this approach, we fabricated a microfluidic device comprised of 5 channels per lane that performs similarly to the 3-channel microfluidic device (Figure **S2**). For these reasons, microfluidic immunoblotting is ideally suited to profile signaling pathways from human samples and other low-protein samples. 

### Monitoring inflammatory pathway activation and protein modifications

**Figure 3:**
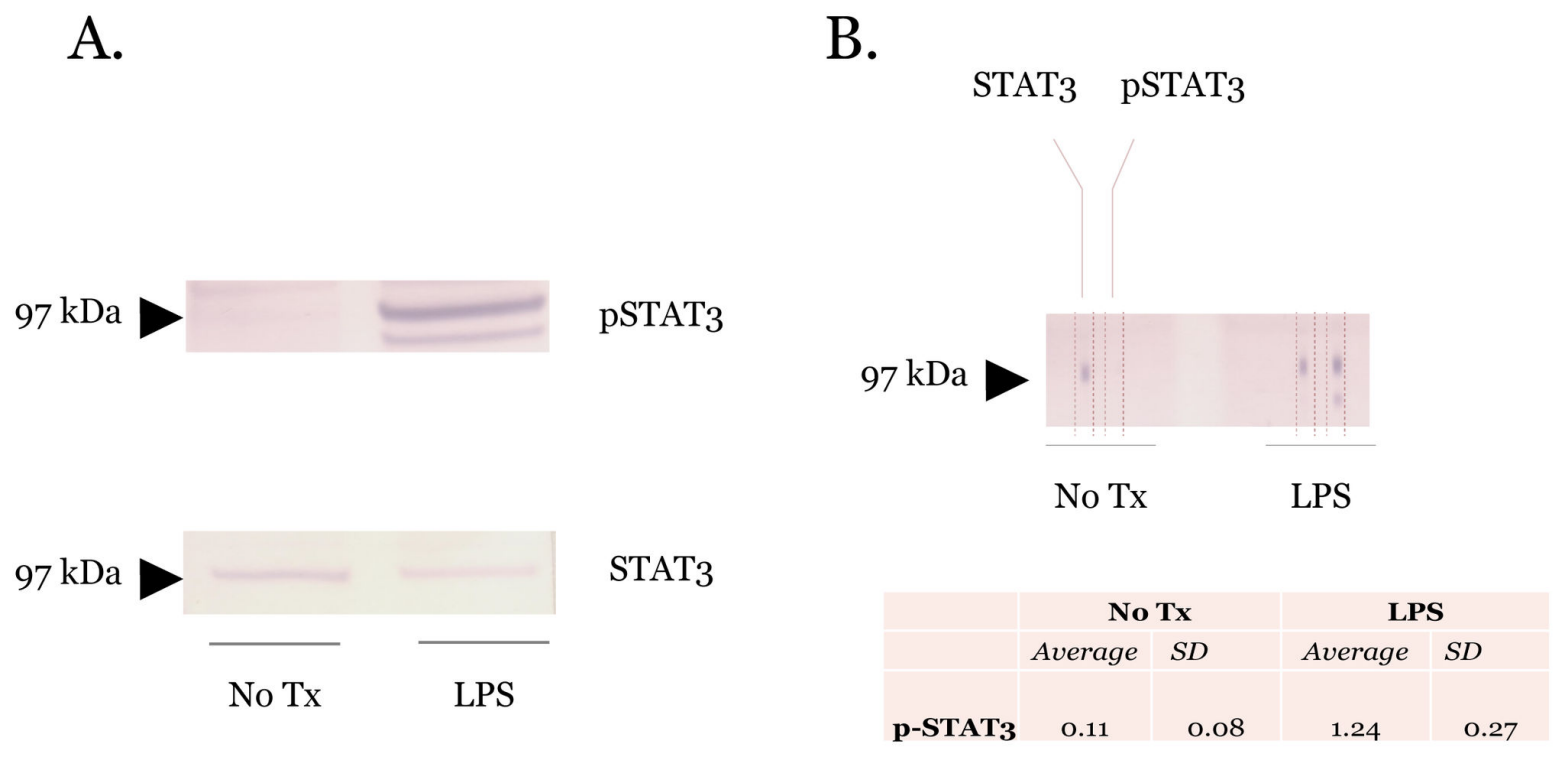
Detection of STAT3 phosphorylation in response to inflammatory stimuli. (A) Traditional immunoblots of RAW264.7 cell lysates showing phosphorylation of STAT3 in response to LPS stimulation. (B) Microfluidic immunoblot on same RAW264.7 cell lysates as (A). The signal intensities from three microfluidic immunoblots were quantified using ImageJ and demonstrate a robust and reproducible signal intensity. The microfluidic device allows for simultaneous monitoring of phospho-STAT3 and STAT3 in the same sample without the need for stripping or reprobing of the PVDF membrane. Immunoblots are representative of at least three independent PVDF membranes.

Inflammatory pathways are often regulated through posttranslational modifications of signaling proteins[13,14]. Monitoring these posttranslational modifications is critical to capture the dynamics of inflammatory responses. Like the NFκB protein family, the signal transducer and activator of transcription (STAT) protein family coordinates many aspects of inflammatory responses[15]. In response to specific cytokines and growth factors, STAT family members are phosphorylated by receptor-associated kinases[16,17]. Protein phosphorylation, a common posttranslational modification, is typically monitored with protein immunoblotting. Because this type of posttranslational modification must be evaluated in the context of the total protein amount, both the phosphorylated and total protein levels must be profiled in parallel. These types of profiling experiments are well suited for microfluidic immunoblotting because the phosphorylated and total protein can be profiled at the same time from the same sample lane. To evaluate this capacity of microfluidic immunoblotting, we stimulated RAW264.7 cells, a macrophage cell line, with lipopolysaccharide (LPS) (100 ng/ml) for 24 hours. After 24 hours, the cell lysates were collected and probed for phospho-STAT3 and STAT3 with both microfluidic and traditional protein immunoblotting. Similar to traditional immunoblotting, microfluidic immunoblotting detected phospho-STAT3 in LPS stimulated macrophages consistent with inflammatory activation of the STAT pathway (Figure **3**). These results suggest that microfluidic immunoblotting can be used to monitor phosphorylation and other posttranslational modifications. Importantly, the ability to monitor multiple proteins and protein modifications greatly improves assay throughput and reduces sample requirements without sacrificing data fidelity. 

### Monitoring multicomponent, inflammatory pathways

**Figure 4:**
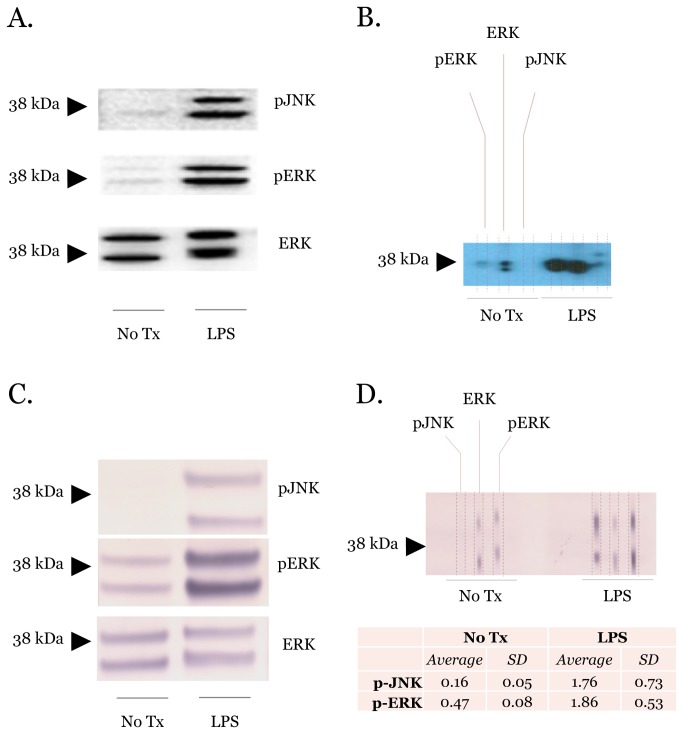
Protein immunoblots of the MAPK pathway using different chemiluminescent detection modalities. (A) Traditional immunoblots of RAW264.7 cell lysates with or without LPS for 45 minutes. Membranes were probed for phospho-JNK and phospho-ERK using total ERK as the loading control. Primary antibodies were detected using HRP chemiluminescent techniques. (B) Microfluidic immunoblots mirroring conditions in (A). (C) and (D) are identical to (A) and (B) except they were performed using AP chemiluminescent secondary antibodies to detect primary antibodies. The signal intensities from three microfluidic immunoblots were quantified using ImageJ. Immunoblots are representative of at least three independent PVDF membranes.

The mitogen activated protein kinase (MAPK) pathway is another pathway that coordinates inflammatory responses[18]. MAPKs are serine/threonine-specific protein kinases that transduce extracellular signals and regulate diverse cellular responses including cell proliferation, differentiation, and apoptosis. One of the difficulties in monitoring this pathway is that it has multiple protein components with different activation properties. These complexities make monitoring the MAPK pathway with traditional immunoblotting time and resource intensive. We hypothesized that some of these complexities could be addressed using microfluidic immunoblotting. To determine the efficacy of the microfluidic device to monitor the MAPK pathway, we stimulated RAW264.7 cells with LPS (100 ng/ml) for 45 minutes and collected cell lysates. We probed for phospho-JNK, phospho-ERK, and total ERK with traditional and microfluidic protein immunoblotting (Figure **4**). Traditional immunoblotting required three different gels and PVDF membranes. In contrast, microfluidic immunoblotting obtained similar results using only two sample lanes from a single gel and PVDF membrane. By simultaneously probing for total ERK from the same sample, we minimized the time and resources compared with traditional immunoblotting. 

We also wanted to evaluate the flexibility of the microfluidic immunoblotting approach with other common chemiluminescent detection methods. Both HRP and AP have excellent sensitivity. However, this higher sensitivity also makes them more susceptible to background noise. We ran a microfluidic immunoblot using the same cell lysates and MAPK antibodies as above. We then used a horseradish peroxidase secondary antibody and substrate to detect the primary antibodies. The efficacy of HRP detection was equivalent to alkaline phosphatase detection using the microfluidic immunoblotting device (Figure **4**) further expanding the utility of this approach. 

## Discussion

In this study, we report the design and evaluation of a microfluidic immunoblotting device to profile inflammatory responses. This approach is unique compared with previously described microfluidic immunoblotting platforms in that it: (1) easily interfaces with conventional protein immunoblotting platforms and can be used with several detection modalities, (2) can detect endogenous proteins and protein modifications from research and human samples, and (3) greatly reduces resource (antibody and sample) requirements and enhances assay throughput. 

Generally, microfluidic immunoblotting approaches can be categorized into two types: those that focus on integrating all of the elements of immunoblotting and those that focus on applying microfluidics only to specific phases of immunoblotting. The former group of microfluidic platforms has obvious advantages and represents the ultimate goal of microfluidic immunoblotting platforms[19–22]. As mentioned above, Herr and colleagues have presented several microfluidic platforms integrating the separation, transfer, and detection steps of protein immunoblotting within a single device. Their methods are fast and enable simultaneous monitoring of several proteins. However, the immediate accessibility to typical molecular biology users may be limited since their focus is on automation and integration of immunoblotting steps. Ciaccio et al. recently presented an approach to monitor EGF receptor signaling using microwestern arrays[23]. Their technique captures the throughput advantages of microarray technology with the detection ability of traditional immunoblotting. This approach is clearly an advance, but does require specialized equipment and extensive sample processing. 

Although microfluidic integration of all the traditional immunoblotting elements is the desired goal, achieving this goal requires advances in microfluidics that also must surpass the ease and reproducibility of existing technologies. An intermediate goal is to use microfluidics for only some elements of protein immunoblotting. This goal has the advantages of greater flexibility and minimal end-user investment. However, to realize these advantages, the microfluidic device must be simple, reproducible, and easily interface with the existing immunoblotting technologies and equipment. Our microfluidic approach most closely resembles that of Pan and colleagues and focuses on integrating microfluidics with the detection phase of protein immunoblotting[11]. Although both approaches use a microfluidic device that interfaces with slab gels, there are several important differences. First, to reduce the complexity of the device, we used a low-volume syringe to load the microfluidic channels to obviate the need for pumps and tubing. Second, to improve the detection limits of our microfluidic device, we used chemiluminescent detection methodologies because of the increased sensitivity compared with fluorescent detection methodologies. Although the increased sensitivity improves detection limits, it requires a microfluidic platform with no leaking and a high degree of stability to maintain a low background signal. Lastly, in contrast to the microfluidic approach by Pan and colleagues, our approach resulted in primary antibody concentrations similar to those of traditional protein immunoblotting leading to greater reductions in primary antibody requirements. 

In conclusion, we have presented the design and evaluation of a microfluidic immunoblotting device for rapid profiling of inflammatory signaling pathways. The design of the microfluidic device allows for the addition of more microfluidic channels that would further increase the amount of proteins that can be simultaneously profiled. The microfluidic approach maintains the data fidelity of traditional protein immunoblotting but greatly improves assay throughput and reduces resource requirements. Additionally, this approach is still compatible with membrane stripping and other PVDF membrane manipulations allowing for further microfluidic profiling of proteins. We anticipate that this microfluidic immunoblotting device will have utility in molecular biology and clinical diagnostics. 

## Supporting Information

Figure S1 **Comparison of antibody and protein signal dependence of traditional and microfluidic protein immunoblotting.** Immunoblots from PBMCs were run and processed as described in the Materials and Methods section. Data are from three independent PBMC immunoblots (from the same sample) for RelA/p65 at four protein concentrations (5, 1, 0.5 and 0.1 µg) and three antibody dilutions. The normalized signal intensities represent measurements from 3 independent immunoblots. Under the conditions tested, the signal intensity was more dependent on protein concentration than on antibody concentration for both traditional and microfluidic protein immunoblotting.
(DOCX)

Figure S2 **Example of microfluidic protein immunoblot generated using a 5-channel per lane microfluidic device.**
(DOCX)
