# Supplementary material for: Profiling Inflammatory Responses with Microfluidic Immunoblotting
Source: PLoS One. 2013 Nov 27;8(11):e81889. doi: 10.1371/journal.pone.0081889 (PMC3842271; doi:10.1371/journal.pone.0081889)
Supplement: Figure S1 — Comparison of antibody and protein signal dependence of traditional and microfluidic protein immunoblotting. Immunoblots from PBMCs were run and processed as described in the Materials and Methods section. Data are from three independent PBMC immunoblots (from the same sample) for RelA/p65 at four protein concentrations (5, 1, 0.5 and 0.1 µg) and three antibody dilutions. The normalized signal intensities represent measurements from 3 independent immunoblots. Under the conditions tested, the signal intensity was more dependent on protein concentration than on antibody concentration for both traditional and microfluidic protein immunoblotting. (DOCX) [file pone.0081889.s001.docx]

**
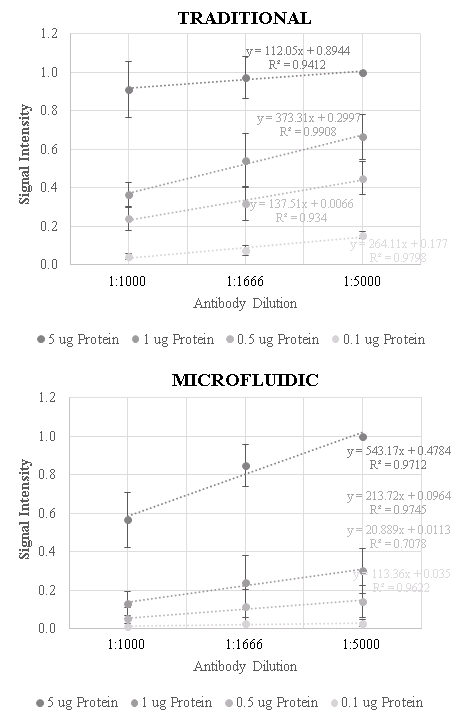
**

**Figure S1**. **Comparison of antibody and protein signal dependence of traditional and microfluidic protein immunoblotting.** Immunoblots from PBMCs were run and processed as described in the Materials and Methods section. Data are from three independent PBMC immunoblots (from the same sample) for RelA/p65 at four protein concentrations (5, 1, 0.5 and 0.1 µg) and three antibody dilutions. The normalized signal intensities represent measurements from 3 independent immunoblots. Under the conditions tested, the signal intensity was more dependent on protein concentration than on antibody concentration for both traditional and microfluidic protein immunoblotting.
