# Supplementary material for: Profiling Inflammatory Responses with Microfluidic Immunoblotting
Source: PLoS One. 2013 Nov 27;8(11):e81889. doi: 10.1371/journal.pone.0081889 (PMC3842271; doi:10.1371/journal.pone.0081889)
Supplement: Figure S2 — Example of microfluidic protein immunoblot generated using a 5-channel per lane microfluidic device. (DOCX) [file pone.0081889.s002.docx]

**
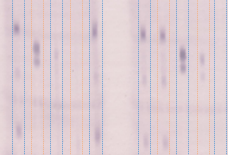
**

**Figure S2**. Example of microfluidic protein immunoblot generated using a 5-channel per lane microfluidic device.
